# Innervation of the Nose and Nasal Region of the Rat: Implications for Initiating the Mammalian Diving Response

**DOI:** 10.3389/fnana.2018.00085

**Published:** 2018-11-13

**Authors:** Paul F. McCulloch, Kenneth A. Lahrman, Benjamin DelPrete, Karyn M. DiNovo

**Affiliations:** Department of Physiology, College Graduate Studies, Chicago College of Osteopathic Medicine, Midwestern University, Downers Grove, IL, United States

**Keywords:** afferent pathways, nasal cavity, nose, trigeminal nerve, spinal trigeminal nucleus, anterior ethmoidal nerve, nasopalatine nerve, diving response

## Abstract

Most terrestrial animals demonstrate an autonomic reflex that facilitates survival during prolonged submersion under water. This diving response is characterized by bradycardia, apnea and selective increases in peripheral vascular resistance. Stimulation of the nose and nasal passages is thought to be primarily responsible for providing the sensory afferent signals initiating this protective reflex. Consequently, the primary objective of this research was to determine the central terminal projections of nerves innervating the external nose, nasal vestibule and nasal passages of rats. We injected wheat germ agglutinin (WGA) into specific external nasal locations, into the internal nasal passages of rats both with and without intact anterior ethmoidal nerves (AENs), and directly into trigeminal nerves innervating the nose and nasal region. The central terminations of these projections within the medulla were then precisely mapped. Results indicate that the internal nasal branch of the AEN and the nasopalatine nerve, but not the infraorbital nerve (ION), provide primary innervation of the internal nasal passages. The results also suggest afferent fibers from the internal nasal passages, but not external nasal region, project to the medullary dorsal horn (MDH) in an appropriate anatomical way to cause the activation of secondary neurons within the ventral MDH that express Fos protein during diving. We conclude that innervation of the anterior nasal passages by the AEN and nasopalatine nerve is likely to provide the afferent information responsible for the activation of secondary neurons within MDH during voluntary diving in rats.

## Introduction

Aquatic, semi-aquatic and even primarily terrestrial animals (including humans) demonstrate an autonomic reflex that enables survival during prolonged submersion under water. This so-called diving response is characterized by bradycardia, apnea, and a selective increase in peripheral vascular resistance causing a redistribution of blood flow to maintain perfusion of the heart and brain while limiting flow to non-exercising muscles (Butler and Jones, 1997; Panneton, 2013). These cardiorespiratory changes occur in response to submersion of the head underwater, but not to swimming on the water surface (McCulloch et al., 2010; Panneton et al., 2010b). Primary afferent fibers innervating the paranasal area and anterior nasal mucosa are important for the initiation of this autonomic reflex (McCulloch, 2012; Panneton, 2013). The specific stimulus that activates this reflex is unknown, however it may possibly involve activation of chemesthetic trigeminal chemoreceptors (Finger et al., 2003; Munger et al., 2009; Silver and Finger, 2009; Tizzano and Finger, 2013).

The nasociliary nerve, a branch of the ophthalmic division of the trigeminal nerve, becomes the anterior ethmoidal nerve (AEN) as it exits the orbit through the ethmoidal foramen (Greene, 1963; Shankland, 2001a; Prendergast, 2013; Gray et al., 2016). Electrical stimulation of the AEN produces an intense cardiorespiratory reflex similar to the diving response (Drummond and Jones, 1979; Dutschmann and Herbert, 1998; McCulloch et al., 1999; Rozloznik et al., 2009). Additionally, acute bilateral sectioning of the AEN severely attenuates the cardiorespiratory response to nasal stimulation in anesthetized rats (Rybka and McCulloch, 2006). Consequently the AEN, and all its branches that innervate the nose and nasal passages, is thought to be primarily responsible for providing the sensory afferent signals that initiate the protective cardiorespiratory changes seen in response to nasal stimulation (McCulloch, 2012; Panneton, 2013). Central projections of the AEN terminate primarily within the spinal trigeminal nucleus caudalis, also known as the medullary dorsal horn (MDH; Panneton, 1991; Panneton et al., 2006; Hollandsworth et al., 2009). The ventral tip of MDH contains secondary neurons that receive afferent information from the AEN (Hollandsworth et al., 2009). These secondary trigeminal neurons become activated, as indicated by the production of Fos protein (Coggeshall, 2005), during voluntary diving in rats. After repetitive voluntary diving Fos-positive neurons are located within the ventral tip of MDH, between pyramidal decussation and obex, peaking just rostral to calamus scriptorius (McCulloch, 2005; Panneton et al., 2012; McCulloch et al., 2016). Thus the ventral tip of MDH serves as the initial afferent node in the neural circuitry of the diving response.

More recent research, however, indicates conscious voluntarily diving rats retain the cardiorespiratory responses to diving 7 days after bilateral sectioning of the AENs (Chotiyanonta et al., 2013). The cardiovascular responses to diving are almost identical in rats with intact AENs compared to rats with bilaterally sectioned AENs (Chotiyanonta et al., 2013). This indicates loss of AEN functioning does not eliminate the cardiorespiratory responses occurring during voluntary diving. There are no differences in Fos expression within the ventral MDH during voluntarily diving in rats with or without intact AENs (McCulloch et al., 2016). In addition, after the AENs are cut bilaterally, the number of Fos-positive neurons within the ventral MDH and paratrigeminal nucleus increases significantly in voluntarily diving rats compared with non-diving control rats (McCulloch et al., 2016). Furthermore, there are no significant differences in activation of brainstem areas involved in cardiorespiratory functioning in rats diving with or without intact AENs (McCulloch et al., 2016). These findings indicate the brainstem areas responsible for both afferent and efferent aspects of the diving response are still activated even when they do not receive sensory afferent information via the AEN during submersion under water.

One potential explanation for all these findings is that other nerve(s) innervating the nose and nasal passages, besides the AEN, can be stimulated by underwater submergence. These other nerve(s) send this alternate sensory input centrally, and this afferent information then activates secondary neurons that are part of the afferent limb of the diving response neural circuitry. Additionally, activation of these secondary neurons must be appropriately sufficient to enable activation of the rest (i.e., the integrative and efferent aspects) of the brainstem diving response circuitry. However, this explanation hinges on the assumption these other nasal nerve(s) project to the same location as does the AEN, specifically the ventral MDH. Only with central projections similar to the AEN could these other nerve(s) activate the secondary neurons within the ventral MDH and paratrigeminal nucleus that express Fos protein during diving.

While the central projections of intraoral nerves of the rat are well known (Hamilton and Norgren, 1984; Shigenaga et al., 1986; Takemura et al., 1987, 1991; Hayakawa et al., 2001), the central projections of intranasal innervation of the rat have not been as thoroughly investigated. Consequently, the primary objective of this research was to determine the central terminal projections of nerves innervating the external nose, nasal vestibule, and nasal passages of rats. We injected the transganglionic tracer wheat germ agglutinin (WGA) into specific external nasal locations, into the internal nasal passages, and directly into nerves innervating the nose and nasal region. The central terminations of these projections within the medulla were then precisely mapped. The pattern of WGA labeling from the nasal passages, both from rats with or without intact AENs, were compared to the WGA labeling patterns from specific trigeminal nerves. Given the trigeminal nerve has a three-dimensional pattern of somatotopic organization (Tracey, 1985; Shigenaga et al., 1986; Shankland, 2000), are there other trigeminal nerve branches which also project to the same MDH locations as the AEN, and which could be involved in initiation of the diving response?

## Materials and Methods

All protocols and procedures were approved by the Midwestern University (MWU) Institutional Animal Care and Use Committee (IACUC). This study was carried out in accordance with the recommendations of the NIH for the care and use of laboratory animals. Male Sprague-Dawley rats were obtained from a commercial vendor (Harlan/Envigo, Indianapolis, IN, USA). Animals were kept in pairs, except after tracer injection or surgery (see below) when they were caged singly. All rats were allowed food and water *ad libitum* and were housed in accordance with NIH guidelines for the care and use of laboratory animals.

### Application of WGA

To determine afferent innervation of the nose and nasal passages, the transganglionic tracer WGA was used in three ways. First, WGA was injected into the nasal passages; second, WGA was injected subdermally into specific locations on the external nose; and third, WGA was directly injected into specific nerves known to innervate the nose and nasal region (AEN, nasopalatine nerve, infraorbital nerve (ION), and supratrochlear nerve). All rats were anesthetized with ketamine/xylazine (80 mg·kg^−1^/10 mg·kg^−1^ i.p.), with supplemental dosages given as needed. Immediately before WGA injection, some rats received bilateral AEN sectioning (see below).

#### Injection of WGA Into Nasal Passages and External Nasal Region

**Figure 1:**
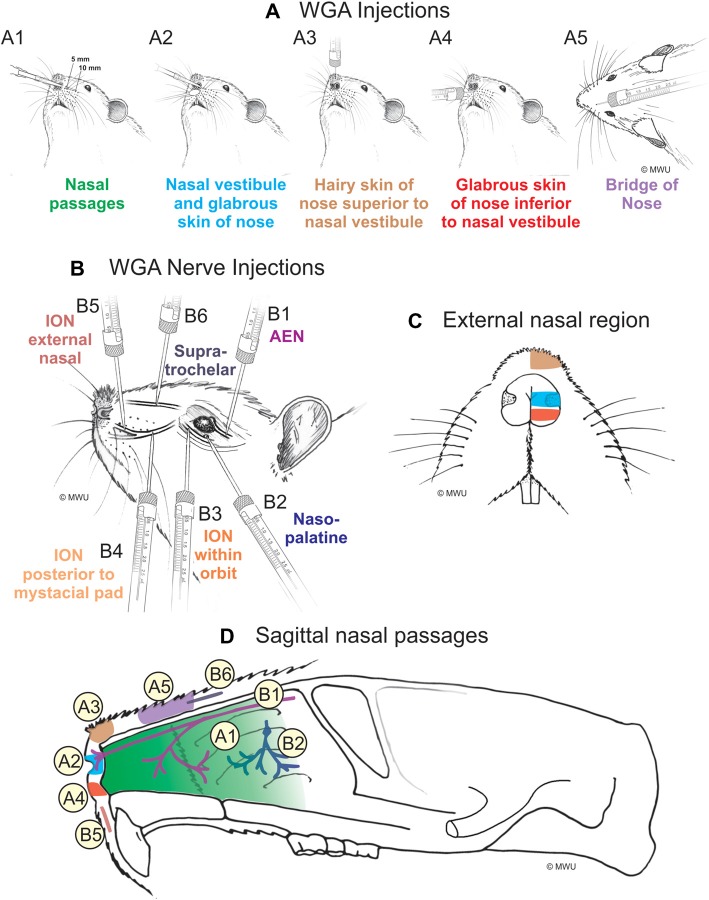
Wheat germ agglutinin (WGA) injection sites into left nose and nasal regions. **(A)** Diagram showing locations of WGA injections in and around nasal region: **(A1)** 5 and 10 mm deep into the nasal passages; **(A2)** immediately inside the left nasal vestibule and brushed on the glabrous skin of the left nostril immediately distal to the nasal vestibule; **(A3)** into the hairy skin of the nose immediately superior to the left nasal vestibule; **(A4)** into the glabrous skin of the nose immediately inferior to the left nasal vestibule; and **(A5)** under skin along the bridge of the nose midway between the nose and the left eye. **(B)** Diagram showing locations of WGA injections directly into nerves: **(B1)** into the left anterior ethmoidal nerve (AEN) within the orbit; **(B2)** into the left nasopalatine nerve as it exited the orbit through the sphenopalatine foramen; **(B3)** into the left infraorbital nerve (ION) as it traversed the infraorbital groove deep within the orbit; **(B4)** into the left ION immediately posterior to the mystacial pad; **(B5)** into the left external nasal nerve immediately distal to the nasal vestibule, and **(B6)** into the left supratrochlear nerve midway between the nose tip and the left eye. **(C)** Summary of WGA injection sites into external nasal regions. Color-coded identification of WGA injection sites follows that from panel **(A)**. **(D)** Summary of WGA injection sites in a sagittal view of internal nasal passages and external nasal region. Color-coded identification of WGA injection sites follows that from panels **(A,B)**.

WGA was placed in five separate locations (Figure 1A; Table 1): (1) WGA (50% WGA (Vector L-1020) and 50% methylcellulose gel (Sigma M7140)) was introduced into the nasal passages (Figure 1A1), following the procedures described by Anton and Peppel (1991). WGA (60 μl total) was injected into the left nasal passages (30 μl each at depths of 5 and 10 mm). In some rats, the AENs were intact (*N*_I_ = 4) and in other rats the AENs were cut bilaterally (*N*_C_ = 4; see below); (2) WGA (50% WGA and 50% methylcellulose gel) was introduced immediately inside the left nasal vestibule and brushed on the glabrous skin of the left nostril immediately distal to the nasal vestibule (20 μl total; *N*_I_ = 3, *N*_C_ = 4; Figure 1A2); (3) WGA (100%) was injected into the hairy skin of the nose immediately superior to the left nasal vestibule (4–10 μl; *N*_I_ = 6; Figure 1A3); (4) WGA (100%) was injected into the glabrous skin of the nose immediately inferior to the left nasal vestibule (4–10 μl; *N*_I_ = 3, *N*_C_ = 4; Figure 1A4); and (5) WGA (100%) was injected under skin along the bridge of the nose midway between the nose and left eye (10 μl; *N*_I_ = 4, *N*_C_ = 4; Figure 1A5).

**Table 1:** Summary of the number of rats used for injection of wheat germ agglutinin (WGA) into nasal passages and external nasal region.

WGA injection site	AEN status	
	AENs intact (*N*)	AENs cut bilaterally (*N*)
Nasal passages (Figure 1A1)	4	4
Nasal vestibule and glabrous	3	4
skin of nose (Figure 1A2)
Hairy skin of nose superior to	6	-
nasal vestibule (Figure 1A3)
Glabrous skin of nose inferior to	3	4
nasal vestibule (Figure 1A4)
Bridge of nose (Figure 1A5)	4	4

#### Injection of WGA Into Nerves Innervating the Nasal Region

WGA (100%) was injected into four trigeminal nerve branches (Figure 1B; Table 2). (1) WGA (1 μl) was injected directly into the left AEN as it traversed the orbit (*N* = 4; Figure 1B1). (2) WGA was injected directly into the left nasopalatine nerve, as it exited the orbit through the sphenopalatine foramen deep to the main bundle of the ION within the orbit (*N* = 8; Figure 1B2). WGA injection into the nerve (2.5 μl) was followed by placing a small WGA-soaked cotton ball directly on the nerve for 15 min. This WGA application procedure was performed twice for each rat. (3) WGA (2 μl) was injected directly into three different locations of the left ION: (i) the main ION bundle as it traversed the infraorbital groove deep within the orbit, distal to the exit of the nasopalatine nerve, but proximal to the infraorbital fissure (*N* = 4; Figure 1B3); (ii) a branch of the left ION immediately posterior to the mystacial pad before its terminal branching into the external nasal and superior labial nerves (*N* = 4; Figure 1B4). This nerve branch separates from the ION before it reaches the mystacial pad; and (iii) the external nasal nerve, a small terminal ION branch innervating the glabrous skin of the nose immediately distal to the nasal vestibule (*N* = 5; Figure 1B5). To access the ION for injection sites **ii** and **iii**, an incision was made on the left cheek between the anterior tip of the eye and the second and third rows of the mystacial pad. These ION branches were accessed deep to the highly myelinated branches of the ION innervating the whiskers; and (4) WGA (2.5 μl) was injected into the left supratrochlear nerve, midway between the nose tip and the left eye (*N* = 8; Figure 1B6).

**Table 2:** Summary of the number of rats used for injection of WGA into nerves innervating the nasal region.

WGA injection site	*N*
AEN (Figure 1B1)	4
Nasopalatine nerve (Figure 1B2)	8
ION within orbit (Figure 1A3)	4
ION posterior to mystacial pad (Figure 1B4)	4
ION external nasal (Figure 1B5)	5
Supratrochlear (Figure 1B6)	8

#### AEN Surgery

After a dorsal skin incision, the AEN was accessed within the orbit by laterally retracting orbital contents. Technically, within the orbit the AEN is the nasociliary nerve; the nasociliary nerve becomes the AEN only after exiting the orbit through the anterior ethmoidal foramen. In all animals receiving AEN sectioning, a 1 mm piece of the AEN was excised and produced for inspection to confirm the denervation. All rats receiving AEN sectioning were given the analgesic Ketoprofen (5 mg·kg^−1^, s.c.) immediately following surgery and again 24 h later.

#### Immunohistological Processing

Following 4–5 days of WGA transport, all rats were euthanized using an intraperitoneal injection of concentrated (26%) sodium pentobarbital. This was followed by ex-sanguination and transcardiac perfusion with saline (0.9% NaCl with 0.25% procaine) and then 4% paraformaldehyde in 0.1 M PBS. The brains of the animals were removed and stored overnight in a buffered paraformaldehyde and sucrose solution. The brainstem was blocked and cut at 50 μm using a freezing microtome. Every third section of the tissue was immunohistologically processed using a fluorescent chromogen for the WGA. Tissue was washed with PBS prior to incubation in 5% BSA for 1 h. The tissue was then incubated overnight in biotinylated goat anti-WGA primary antibody (Vector BA-0024; 15 μl/ml). The next day the tissue was incubated in secondary antibody (Streptavidin DyLight 488; Vector SA-5488: 7 μg/ml) in the dark for 2 h. This procedure produced green fluorescent labeling of the WGA tracer. Individual brainstem sections were arranged into serial order, mounted on glass slides, and coverslipped using hard-set mounting media for fluorescence (Vector H-1500).

### Microscopy and Analysis

All brainstem sections were viewed with a Nikon AR1 confocal microscope, and were compared with a rat brain atlas (Paxinos and Watson, 1998). Analysis consisted of comparing the WGA central projections between rats with different injection locations. Color images of the sections were taken to assist in the analysis of the projection patterns and to visually demonstrate differences viewed during microscopy (NIS Elements Confocal, Melville, NY, USA).

To facilitate comparisons between different labeling patterns, sections were also organized into seven rostral-caudal levels, each approximately 200 μm thick, from the rostral spinal cord to obex, based on Figures 73–78 from Paxinos and Watson (1998). WGA data from all individual brainstem sections were superimposed on the appropriate cross-section, providing representative labeling at each of the seven rostral-caudal levels. The rostral end of the pyramidal decussation represented the transition between the rostral spinal cord and caudal medulla. Calamus scriptorius was defined as the most caudal extent of area postrema, just as the dorsal columns separated. Obex was defined as the opening of the central canal into the fourth ventricle. All graphs were created using SigmaPlot (SYSTAT, San Jose, CA, USA). Figures were composed and labeled using CorelDRAW (Corel, Ottawa, ON, Canada).

## Results

### Application of WGA

#### Injection of WGA Into Nasal Passages

**Figure 2:**
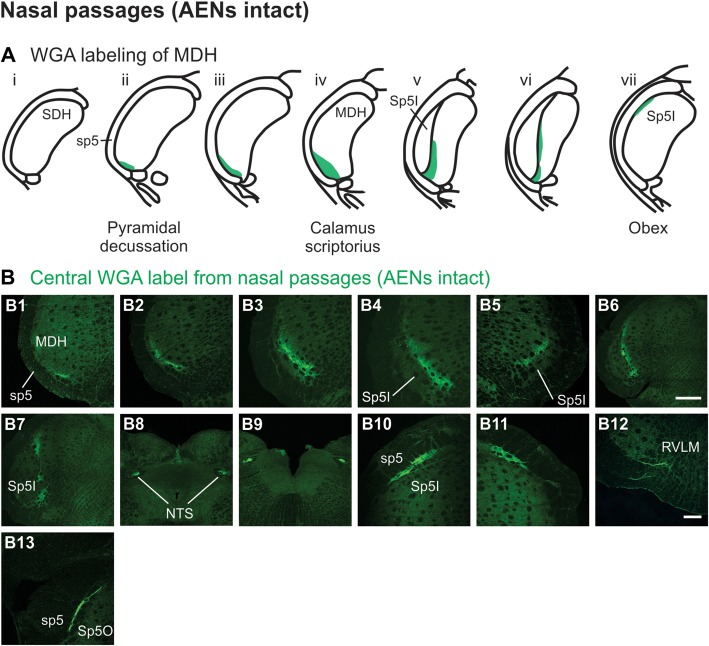
WGA label from nasal passages after WGA was injected into the left nasal passages with AENs intact (see Figure 1A1). **(A)** Summary cross-sections showing WGA labeling of medullary dorsal horn (MDH). **(B)** Photomicrographs showing central labeling within the brainstem. Label was found caudally in the ventral tip of the ipsilateral superficial MDH **(B1–B3)**. As Sp5I began to appear ventrolaterally, label within MDH was found more medially both ipsilaterally **(B4,B6,B7)** and contralaterally **(B5)**. Label was also found within NTS **(B8,B9)**. Rostral to obex label was found bilaterally along the dorsolateral edge of Sp5I **(B10,B11)**. Just caudal to the facial nucleus, label extended from the ventral tip of the trigeminal tract into the rostral ventrolateral medulla **(B12)**. More rostrally, label was found as a thin strip in the dorsolateral trigeminal tract adjacent to spinal trigeminal nucleus oralis **(B13)**. Rostral-caudal orientation: pyramidal decussation: caudal to **(B1)**; calamus scriptorius: **(B3)**; obex: rostral to **(B7,B9)**. Scale bar in **(B6)** for **(B6–B9)** = 500 μm. Scale bar in **(B12)** for all other panels = 200 μm.

After injection of tracer 5–10 mm into the left nasal passages (Figure 1A1) in rats with intact AENs, WGA was found primarily within the ipsilateral MDH between the pyramidal decussation and obex (Figures 2A,B). At the most caudal levels of the brainstem, just rostral to the pyramidal decussation, label was found only in the ventral tip of the ipsilateral superficial laminae (laminae I and II) of MDH (Figure 2B1). This label extended into a more dorsolateral-ventromedial orientation when moving rostrally toward calamus scriptorius (Figures 2B2,B3). No label was found in deeper MDH locations (laminae III, IV and V). As Spinal Trigeminal Nucleus Interpolaris (Sp5I) began to appear ventrolaterally at calamus scriptorius, label within the left MDH was found more medially (Figure 2B4). Label at this level was also found contralaterally within the right MDH (Figure 2B5). As Sp5I became more prominent, label within MDH was found more medially and in a dorsal-ventral orientation, and began to break up (Figures 2B6,B7). Label was also found either ipsilaterally or bilaterally within nucleus tractus solitarius (NTS), depending upon the animal. NTS label was located primarily within the interstitial subnucleus, and extended from caudal to calamus scriptorius (Figure 2B8; corresponding to rostral-caudal level of Figure 2B2) to rostral to obex (Figure 2B9). At and just rostral to obex label was found bilaterally along the dorsolateral edge of Sp5I adjacent to the trigeminal tract (Figures 2B10,B11). Just caudal to the facial nucleus, label extended from the ventral tip of the trigeminal tract into the rostral ventrolateral medulla (RVLM; i.e., C1 and Bötzinger area; Figure 2B12). More rostrally, label was found ipsilaterally, but in some animals bilaterally, as a thin strip in the dorsolateral trigeminal tract adjacent to spinal trigeminal nucleus oralis (SP5O; Figure 2B13).

**Figure 3:**
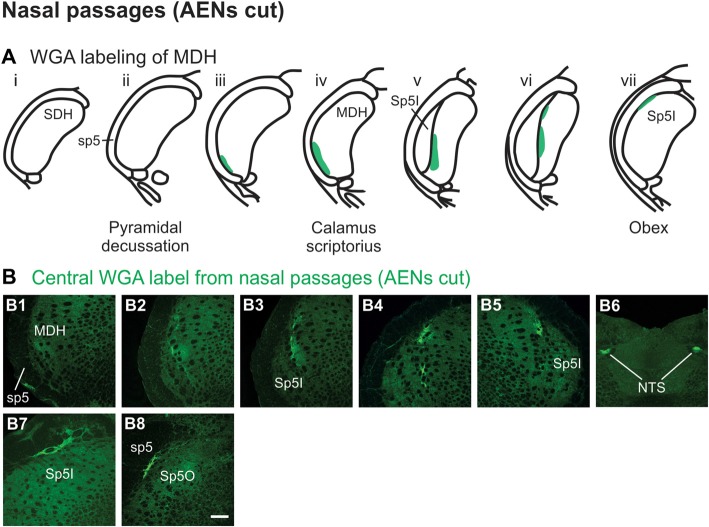
WGA label from nasal passages after WGA was injected into the left nasal passages with AENs cut bilaterally (see Figure 1A1). **(A)** Summary cross-sections showing WGA labeling of MDH. **(B)** Photomicrographs showing central labeling within the brainstem. Label was found caudally within the ipsilateral superficial MDH **(B1,B2)**. As Sp5I appeared ventrolaterally, labeling of the dorsal MDH was found both ipsilaterally **(B3,B4)** and contralaterally **(B5)**. Label was also found within NTS **(B6)**. Rostral to obex label was found ipsilaterally along the dorsolateral edge of Sp5I **(B7)**. More rostrally, label was found bilaterally as a thin strip in the dorsolateral trigeminal tract adjacent to the spinal trigeminal nucleus oralis **(B8)**. Rostral-caudal orientation: calamus scriptorius: rostral to **(B1)**; obex: rostral to **(B4,B5)**. Scale bar in **(B8)** = 200 μm.

Injection of WGA tracer 5–10 mm into the left nasal passages (Figure 1A1) of rats with bilaterally sectioned AENs (Figures 3A,B) produced a somewhat similar pattern of labeling within the brainstem compared to when the AEN was intact. Label was found ventrally within the ipsilateral superficial laminae (laminae I and II) of MDH near to calamus scriptorius (Figures 3B1,B2). Compared to animals with intact AENs, label typically extended neither as far caudally nor into the extreme ventral tip of MDH. As Sp5I appeared laterally, ipsilateral labeling of the dorsal MDH, but not the ventral MDH, was found (Figures 3B3,B4). Label was also found in the contralateral dorsal MDH at this level (Figure 3B5). Unilateral or bilateral labeling of the interstitial NTS near calimus scriptorius, depending upon the animal, still remained (Figure 3B6; similar to Figure 2B8). Another difference in the pattern of labeling was no label extended into the RVLM just caudal to the facial nucleus (compare to Figure 2B12). Just rostral to obex, label was found ipsilaterally along the dorsolateral edge of Sp5I adjacent to the trigeminal tract (Figure 3B7; similar to Figure 2B10). More rostrally, label was also found bilaterally in most animals as a thin strip in the trigeminal tract adjacent to the dorsolateral SP5O (Figure 3B8; similar to Figure 2B13).

**Figure 4:**
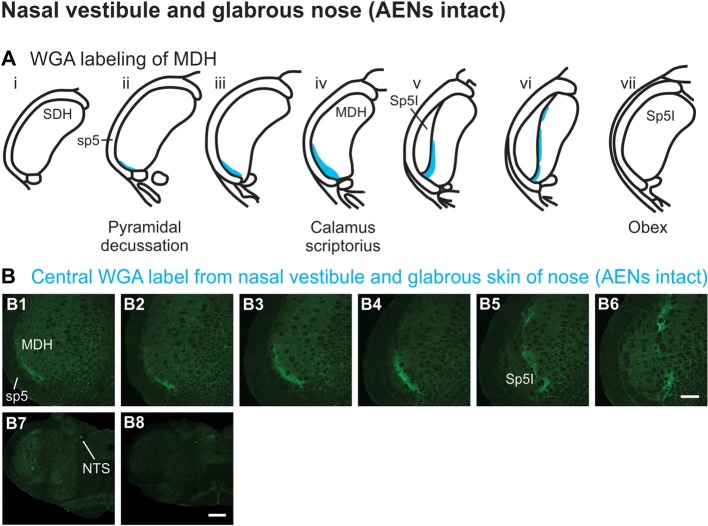
WGA label from nasal vestibule after WGA introduced immediately inside the left nasal vestibule and brushed on the glabrous skin of the left nostril immediately distal to the nasal vestibule with AENs intact (see Figure 1A2). **(A)** Summary cross-sections showing WGA labeling of MDH. **(B)** Photomicrographs showing central labeling within the brainstem. Label was found caudally in the ventral tip of ipsilateral superficial MDH **(B1–B3)**. As Sp5I appeared ventrolaterally, label within MDH was more medial **(B4–B7)**. Label was also found within NTS **(B7)**. When tracer was injected into these same locations in animals with bilaterally sectioned AENs, no label was found within the ventral MDH but was found within NTS **(B8)**. Rostral-caudal orientation: pyramidal decussation: caudal to **(B1)**; calamus scriptorius: **(B4)** and **(B8)**; obex: immediately rostral to **(B7)**. Scale bar in **(B8)** for **(B7)** and **(B8)** = 500 μm. Scale bar in **(B6)** for all other panels = 200 μm.

WGA tracer was injected immediately inside the left nasal vestibule and brushed superficially on the glabrous skin immediately outside the left nasal vestibule (Figure 1A2) in rats with intact AENs. Label was found primarily within the ventral tip of the ipsilateral superficial laminae of MDH (Figures 4A,B). Rostral to the pyramidal decussation, label was found only in the ventral tip of MDH (Figures 4B1–B3). As Sp5I began to appear ventrolaterally, label within MDH was more medial (Figures 4B4–B7), remaining at the ventral tip but also appearing dorsally at more rostral levels (Figures 4B5–B7). Label was also found within the ipsilateral interstitial NTS (Figure 4B7). When tracer was injected into these same locations in animals with bilaterally sectioned AENs, no label was found within the ventral MDH (Figure 4B8). However, some label was found in the ipsilateral interstitial and dorsolateral NTS at the level of calamus scriptorius (Figure 4B8).

#### Injection of WGA Into the External Nasal Region

**Figure 5:**
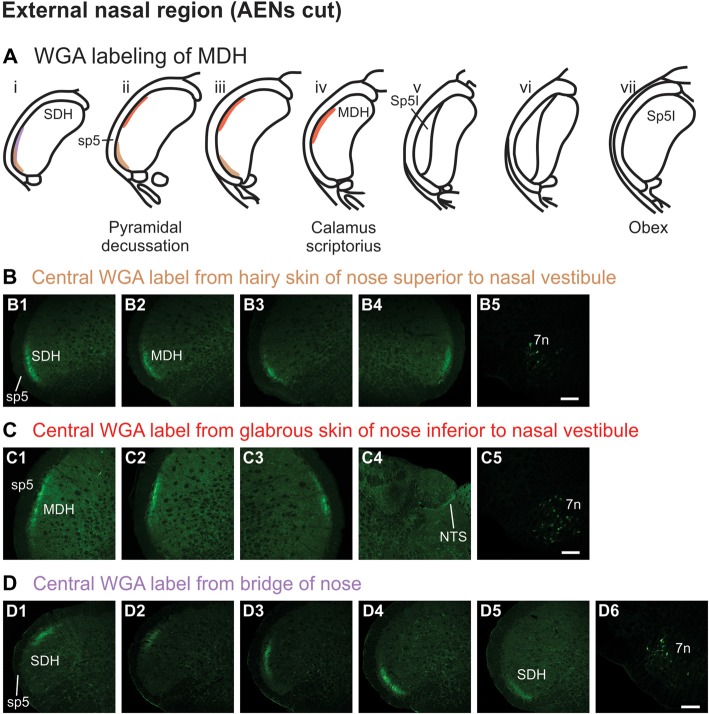
WGA label from external nasal region with AENs cut bilaterally. **(A)** Summary cross-sections showing WGA labeling of MDH. **(B)** Photomicrographs showing central labeling within the brainstem after WGA was injected into the hairy skin of the nose immediately superior to the left nasal vestibule (see Figure 1A3). Label was found within the ventral tip of the superficial laminae of the spinal and medullary dorsal horn both ipsilaterally **(B1–B3)** and contralaterally **(B4)**. Label was found within the ipsilateral 7n **(B5)**. Rostral-caudal orientation: pyramidal decussation: **(B2)**. Scale bar in **(B5)** for all panels = 200 μm. **(C)** Photomicrographs showing central labeling within the brainstem after WGA was injected into the glabrous skin of the nose immediately inferior to the left nasal vestibule (see Figure 1A4). Label was found within the superficial laminae of the lateral and dorsolateral MDH both ipsilaterally **(C1,C2)** and contralaterally **(C3)**. Label was found along the edge of the ipsilateral dorsolateral NTS **(C4)**, and ipsilateral 7n **(C5)**. Rostral-caudal orientation: pyramidal decussation: caudal to **(C1)**; calamus scriptorius: **(C2)**. Scale bar in **(C5)** for all panels = 200 μm. **(D)** Photomicrographs showing central labeling within the brainstem after WGA was injected under the hairy skin along the bridge of the nose, left of the midline between the nose and left eye (see Figure 1A5). Label was found within the superficial laminae of the dorsolateral SDH **(D1)**, lateral SDH **(D2,D3)**, and ventrolateral tip of SDH **(D4,D5)**. Label was found within the ipsilateral 7n **(D6)**. Rostral-caudal orientation: pyramidal decussation: rostral to **(D5)**. Scale bar in **(D6)** for all panels = 200 μm.

WGA tracer was injected into the hairy skin of the tip of the nose superior to the left nasal vestibule (Figure 1A3) only in rats with bilaterally sectioned AENs. Label was found primarily within the ventral tip of the superficial laminae of the spinal and MDH (Figures 5A,B). Label was both ipsilateral (Figures 5B1–B3) and contralateral (Figure 5B4). Label was found primarily caudal to the pyramidal decussation within the spinal dorsal horn (SDH). Within MDH, label ended before reaching calamus scriptorius at its rostral extreme. No label appeared at levels containing Sp5I, nor did label appear in NTS. Label was found within the ipsilateral 7n (Figure 5B5).

WGA tracer was injected into the glabrous skin of the nose inferior to the left nasal vestibule in rats with bilaterally sectioned AENs (Figure 1A4). Label was found within the superficial laminae of the lateral and dorsolateral MDH between the pyramidal decussation and calamus scriptorius (Figures 5A,C). Label was both ipsilateral (Figures 5C1,C2) and contralateral (Figure 5C3). At the level of area postrema, label was not found in MDH but was along the edge of the ipsilateral dorsolateral NTS (Figure 5C4). Label was found within the ipsilateral 7n (Figure 5C5). When WGA tracer was placed in this same location in animals with intact AENs, the labeling pattern was nearly identical compared to when the AEN was bilaterally sectioned (not shown).

WGA tracer was injected under the skin along the left side of the bridge of the nose (Figure 1A5) in rats with bilaterally sectioned AENs. Label was found only caudal to the pyramidal decussation and therefore within the spinal cord (Figures 5A,D). Label was primarily ipsilateral (Figures 5D1–D5), but also contralateral (not shown). At its most caudal extreme, label was found within the superficial laminae of the dorsolateral SDH (Figure 5D1). Slightly rostrally, this label appeared more laterally (Figures 5D2,D3). In its most rostral extreme, but still within the spinal cord, label appeared in the ventrolateral tip of SDH (Figures 5D4,D5). Label was found within the ipsilateral 7n (Figure 5D6). When tracer was placed in this same location in animals with intact AENs, the labeling pattern was nearly identical compared to when the AEN was bilaterally sectioned (not shown).

#### Injection of WGA Into Nerves That Innervate the Nasal Region

**Figure 6:**
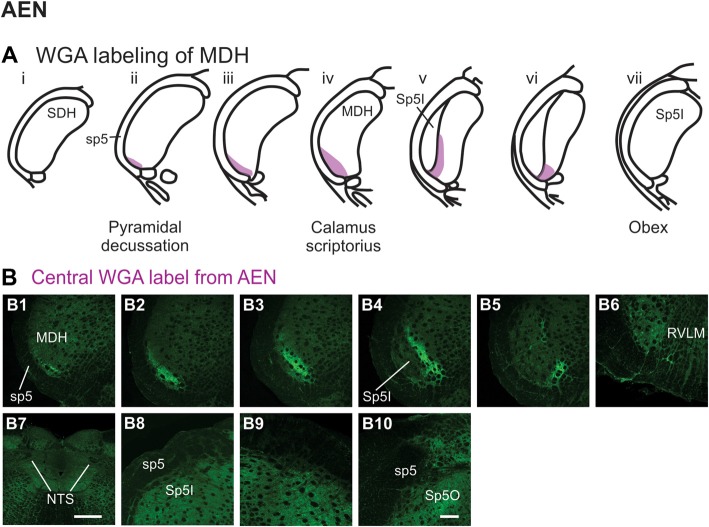
WGA label from AEN (see Figure 1B). **(A)** Summary cross-sections showing WGA labeling of MDH. **(B)** Photomicrographs showing central labeling within the brainstem after WGA was injected into the left AEN. Label was found caudally in the ventral tip of the ipsilateral superficial MDH **(B1–B3)**. As Sp5I appeared ventrolaterally, label within the MDH was more medial **(B4,B5)**. Label extended from the ventral tip of the trigeminal tract into the rostral ventrolateral medulla **(B6)**. No label was found within NTS **(B7)**, along the dorsolateral edge of Sp5I adjacent to the trigeminal tract **(B8,B9)**, or in the trigeminal tract adjacent to the dorsolateral spinal trigeminal nucleus oralis **(B10)**. Rostral-caudal orientation: pyramidal decussation: caudal to **(B1)**; calamus scriptorius: **(B3)**; obex: rostral to **(B5,B8,B9)**. Scale bar in **(B7)** = 500 μm. Scale bar in **(B10)** for all other panels = 200 μm.

After WGA tracer was injected directly into the left AEN (Figure 1B1), label was found primarily within the ventral tip of MDH (Figures 6A,B). At the most caudal levels of the brainstem, just rostral to the pyramidal decussation, label was found only in the ventral tip of the ipsilateral superficial laminae of MDH (Figure 6B1). The amount of label increased when moving rostrally toward calamus scriptorius (Figures 6B2,B3). No label was found in deeper MDH locations. As Sp5I began to appear ventrolaterally, label within the left MDH was found more medially (Figure 6B4). As Sp5I became more prominent, label within MDH was found even more medially, but remained along the ventral edge of interpolaris (Figure 6B5). Label extended from the ventral tip of the trigeminal tract into the RVLM (C1 and Bötzinger area) just caudal to the facial nucleus (Figure 6B6). No label was found contralaterally within the right MDH, within NTS (Figure 6B7), along the dorsolateral edge of Sp5I adjacent to the trigeminal tract (Figures 6B8,B9), or in the trigeminal tract adjacent to the dorsolateral SP5O (Figure 6B10).

**Figure 7:**
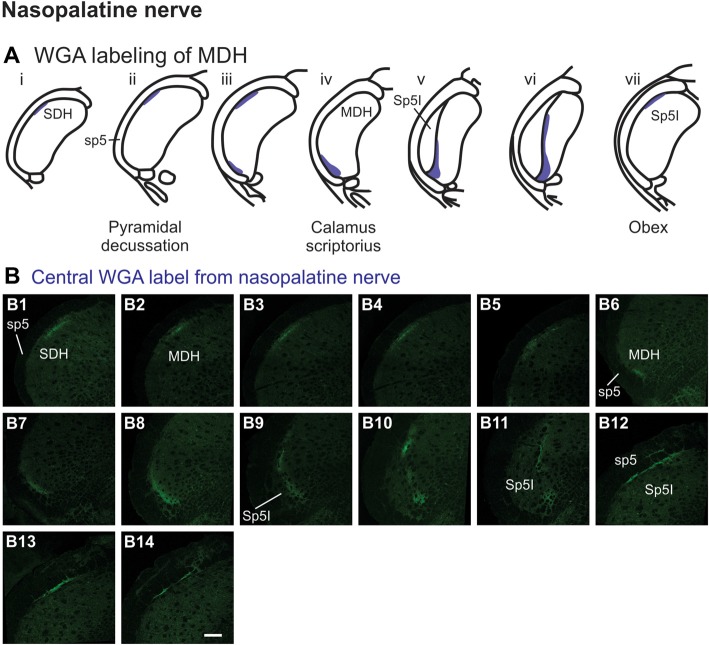
WGA label from nasopalatine nerve (see Figure 1B2). **(A)** Summary cross-sections showing WGA labeling of MDH. **(B)** Photomicrographs showing central labeling within the brainstem after WGA was injected into the left nasopalatine nerve. Three different patterns of labeling were found. The first pattern was found in the caudal ipsilateral dorsolateral superficial MDH **(B1–B5)**. The second pattern was found in the ipsilateral ventrolateral superficial MDH **(B6–B11)**. Label was found caudally in the ventrolateral tip of superficial MDH **(B6–B8)**. As Sp5I appeared ventrolaterally label within MDH was more medial **(B9–B11)**. The third pattern was found ipsilaterally along the dorsolateral edge of Sp5I **(B12–B14)**. Rostral-caudal orientation: for panels **(B1–B5)**—pyramidal decussation: **(B2)**; for panels **(B6–B11)**—calamus scriptorius: **(B7)**; for panels **(B12–B14)**—obex: **(B12)**. Scale bar in **(B14)** for all panels = 200 μm.

After WGA tracer was injected directly into the nasopalatine nerve as it exited the orbit through the sphenopalatine foramen deep to the main bundle of the ION within the orbit (Figure 1B3), three different patterns of labeling were found (Figures 7A,B). The first pattern was found in the ipsilateral caudal dorsolateral superficial MDH (Figures 7B1–B5), but in only two of eight rats. This label first appeared caudal to pyramidal decussation, and extended rostrally to just caudal to calamus scriptorius. The second pattern was found in the ipsilateral ventrolateral superficial MDH (Figures 7B6–B11). This label first appeared caudal to calamus scriptorius, and extended rostrally to just caudal to obex. Caudally label was in the ventrolateral tip of superficial MDH (Figures 7B6–B8). As Sp5I appeared laterally label appeared more medially along the medial edge of interpolaris, and extended in a dorsal-ventral orientation (Figures 7B9–B11). The third pattern was found ipsilaterally along the dorsolateral edge of Sp5I adjacent to the trigeminal tract (Figures 7B12–B14). This label was at and immediately rostral to obex. After nasopalatine nerve injections label was not found in any other area of the brainstem ipsilaterally or contralaterally.

**Figure 8:**
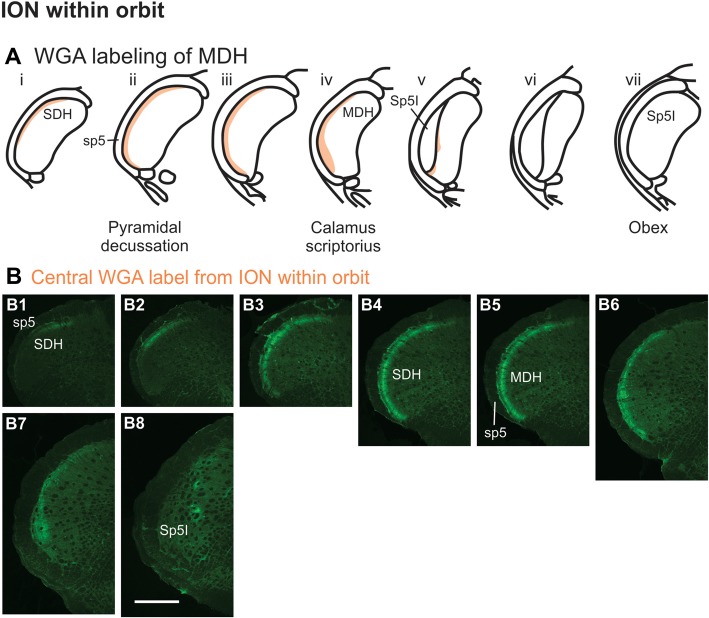
WGA label from orbital ION (see Figure 1B3). **(A)** Summary cross-sections showing WGA labeling of MDH. **(B)** Photomicrographs showing central labeling within the brainstem after WGA was injected into individual branches of the left ION as they passed through the infraorbital groove deep within the orbit. Caudally, within the spinal cord, label was first found more dorsally **(B1,B2)**, but then more laterally **(B3,B4)**. Label was then found throughout the entire dorsal to ventral extreme of the ipsilateral superficial MDH **(B5–B7)**. As Sp5I appeared ventrolaterally, label within MDH was found more medially and dorsally **(B8)**. Rostral-caudal orientation: pyramidal decussation: **(B5)**; calamus scriptorius: **(B7)**; obex: rostral to **(B8)**. Scale bar in **(B8)** for all panels = 500 μm.

After WGA tracer was injected directly into the main ION bundle as it traversed the infraorbital groove deep within the orbit (Figure 1B3), label was found dorsally, laterally and ventrally throughout the ipsilateral superficial laminae of the spinal and MDH (Figures 8A,B). Caudally, within the spinal cord, label was found more dorsally (Figures 8B1,B2), but this shifted more laterally within the rostral spinal cord (Figures 8B3,B4). Label was found throughout the entire dorsal to ventral extreme of the ipsilateral superficial laminae of MDH between the pyramidal decussation caudally (Figure 8B5) and calamus scriptorius rostrally (Figure 8B7). As Sp5I appeared just rostral to calamus scriptorius, label within MDH was found more medially and dorsally (Figure 8B8). No label was found contralaterally within right dorsal horns.

**Figure 9:**
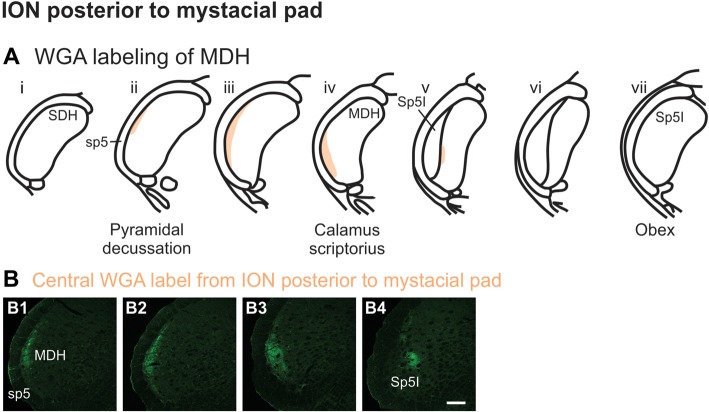
WGA label from ION immediately posterior to the mystacial pad (Figure 1B4). **(A)** Summary cross-sections showing WGA labeling of MDH. **(B)** Photomicrographs showing central labeling within the brainstem after WGA was injected into a branch of the left ION containing both external nasal and superior labial nerves immediately posterior to the mystacial pad. Label was found laterally in the caudal ipsilateral superficial MDH **(B1,B2)**. Label was then found more ventrolaterally **(B3)**. As Sp5I appeared ventrolaterally, label within MDH was found more medially **(B4)**. Rostral-caudal orientation: pyramidal decussation: **(B1)**; calamus scriptorius: **(B4)**; Scale bar in **(B4)** for all panels = 200 μm.

After WGA tracer was injected directly into a branch of left ION immediately posterior to the mystacial pad (Figure 1B4), label was found primarily within the lateral and ventrolateral MDH (Figures 9A,B). At the most caudal levels of the brainstem, just rostral to the pyramidal decussation, label was found laterally in the ipsilateral superficial laminae of MDH (Figure 9B1). Moving rostrally from the pyramidal decussation, label was found more dorsally and ventrally, while still being focused laterally (Figure 9B2). Just caudal to calamus scriptorius, label was found slightly more ventrolaterally (Figure 9B3). As Sp5I began to appear ventrolaterally at the level of calamus scriptorius, label within MDH was found more medially (Figure 9B4). Primary ION projections were not found in any other area of the brainstem ipsilaterally or contralaterally. In two rats label was found in the ipsilateral 7n (not shown).

**Figure 10:**
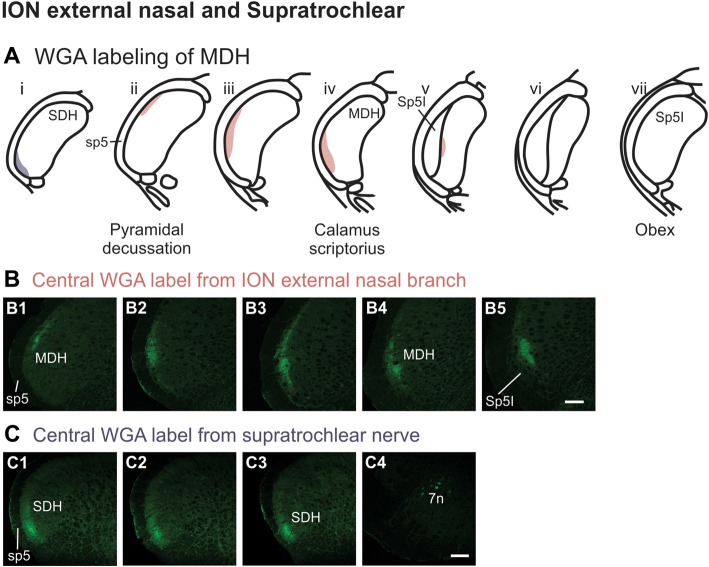
WGA label from external nasal branch of ION and supratrochlear nerve. **(A)** Summary cross-sections showing WGA labeling of MDH. **(B)** Photomicrographs showing central labeling within the brainstem after WGA was injected directly into external nasal branch of the left ION (see Figure 1B5). Caudally label was found dorsolaterally in the ipsilateral superficial MDH **(B1)**. Label then become more lateral **(B2,B3)** and ventrolateral **(B4)**. As Sp5I appeared ventrolaterally, label within MDH was found more medially **(B5)**. Rostral-caudal orientation: pyramidal decussation: caudal to **(B1)**; calamus scriptorius: **(B4)**. Scale bar in **(B5)** for all panels = 200 μm. **(C)** Photomicrographs showing central labeling within the brainstem after WGA was injected into the left supratroclear nerve (see Figure 1B6). Label was found in ventrolateral tip of SDH, approximately 1000 μm caudal to the pyramidal decussation **(C1–C3)**. Label was found within the ipsilateral 7n **(C4)**. Rostral-caudal orientation: pyramidal decussation: approximately 900 μm rostral to **(C3)**. Scale bar in **(C4)** for all panels = 200 μm.

After injection of WGA directly into the external nasal branch of the left ION (Figure 1B5), label was found primarily within the lateral and ventrolateral tip of the MDH (Figures 10A,B). At the most caudal levels of the brainstem, just rostral to the pyramidal decussation, label was found dorsolaterally in the ipsilateral superficial laminae of the MDH (Figure 10B1). When moving rostrally from the decussation, the label tended to become more lateral (Figure 10B2), and then extend more dorsally and ventrally (Figure 10B3). At calamus scriptorius the label was more ventrolateral (Figure 10B4). As Sp5I appeared ventrolaterally, label within MDH was found more medially (Figure 10B5). Primary ION projections were not found in any other area of the brainstem ipsilaterally or contralaterally. In all animals label was found in the ipsilateral 7n (not shown).

After WGA tracer was injected directly into the left supratrochlear nerve (Figure 1B6), label was found in the rostral spinal cord approximately 1,000 μm caudal to the pyramidal decussation (Figures 10A,C). Label was restricted to the ventrolateral tip of the SDH, and only extended for about 500 μm in the rostral-caudal direction (Figures 10C1–C3). More rostrally, label was also found in the ipsilateral 7n (Figure 10C4).

### Summary Composite Figures

**Figure 11:**
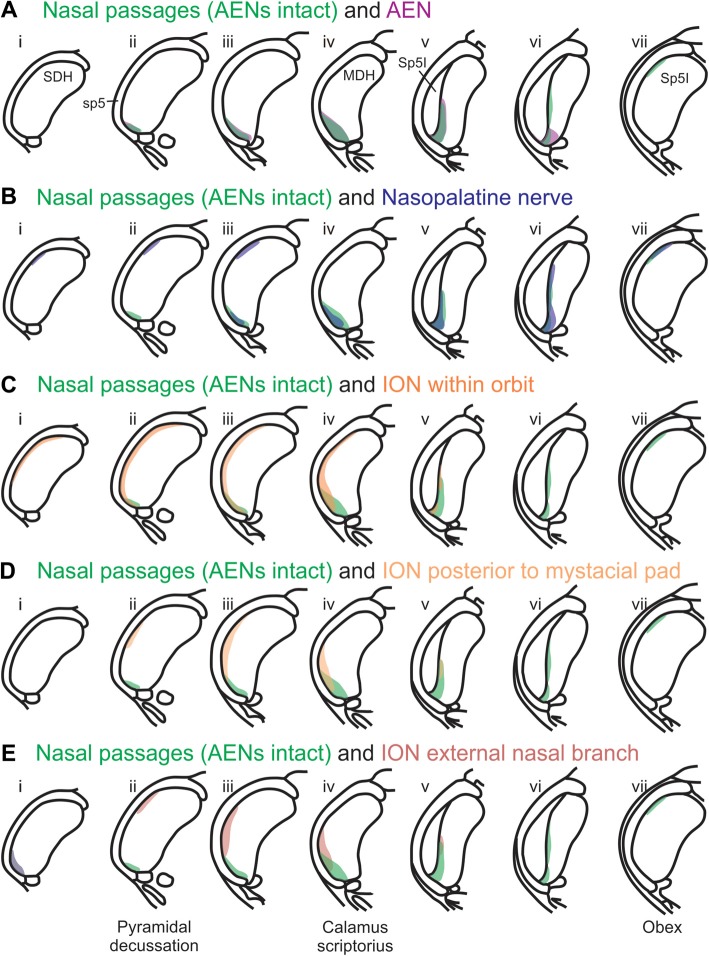
Summary of MDH labeling after WGA injection into the left nasal passages, along with WGA injections into nerves that innervate the left nasal region. Labeling pattern from the nasal passages with the AENs intact (from Figure 2) is shown in transparency overlays with labeling patterns from individual nerves: **(A)** WGA into AEN (from Figure 6); **(B)** WGA into nasopalatine nerve (from Figure 7); **(C)** WGA injection into main ION bundle as it traversed the infraorbital groove deep within the orbit with AENs intact (from Figure 8); **(D)** WGA injection into a branch of ION immediately posterior to the mystacial pad (from Figure 9); and **(E)** WGA injection into external nasal branch of ION (from Figure 10). See Figure 1 for nerve orientation and color coding of WGA injections.

Using the color-coded summary cross-sections showing WGA labeling of the MDH, the labeling pattern from the internal nasal passages (Figure 2) is shown in transparency overlay with the WGA labeling patterns from nerves innervating the nasal region (Figures 6–10). Both the AEN (Figure 11A) and nasopalatine nerve (Figure 11B) show labeling patterns almost completely overlapping the labeling pattern from the internal nasal passages. See “Discussion” section below for further details.

Rostral-caudal orientation: pyramidal decussation: column ii; calamus scriptorius: column iv; obex: column vii. See text for details.

## Discussion

The primary objective of this research was to determine the central terminal projections of nerves innervating the external nose, nasal vestibule, and nasal passages of rats. Using selective injections of the tranganglionic tracer WGA, we found that the central terminal projections of both the AEN and nasopalatine nerve are very similar to the central projections from the internal nasal passages. These central projections were found prominently within the ventral tip of MDH, between pyramidal decussation and obex. In comparison, the central terminal projections of the ION only partially overlap those from the internal nasal passages. Thus both the internal nasal branch of the AEN from the ophthalmic division of the trigeminal nerve, and the nasopalatine nerve from the maxillary division of the trigeminal nerve, are primarily responsible for innervating the internal nasal passages. These findings suggest that both the AEN and nasopalatine nerve may provide afferent innervation important for initiation of the mammalian diving response. The central terminal projections from the external nasal region within the medullary and SDHs suggest that these locations are unlikely to contribute to the initiation of the mammalian diving response. A caveat of this research, however, is that WGA appears to preferentially label small-diameter unmyelinated afferent fibers (Robertson and Grant, 1985; LaMotte et al., 1991).

### Pattern of Central WGA Labeling From the Internal Nasal Passages

After WGA was injected 5–10 mm into the nasal passages, the central tracer terminations were found prominently within the ventral tip of MDH, between pyramidal decussation and obex, especially along the transition with Sp5I just rostral to calamus scriptorius. These present results are similar to those found by Anton and Peppel (1991) in the rat using similar nasal injection techniques. This pattern of labeling within the ventral MDH is also very similar to that found previously when WGA was directly injected into the AEN of rats (Panneton et al., 2006; Hollandsworth et al., 2009), muskrats (Panneton, 1991) and cats (Lucier and Egizii, 1986). Since the AEN labeling almost superimposes upon labeling from the nasal passages (Figure 11A), collectively these findings strongly indicate the internal nasal branch of the AEN provides primary innervation of the internal nasal passages.

#### Differential WGA Labeling of Internal Nasal Passages vs. AEN

There were, however, several differences in the central labeling of the brainstem when WGA was injected into the nasal passages vs. directly into the AEN. First, after injection of WGA into the nasal passages, interstitial NTS was consistently labeled, extending (often bilaterally) from calamus scriptorius to obex. Anton and Peppel (1991) also found NTS labeling after nasal WGA injections. However NTS labeling was not found when WGA was directly injected into the AEN, a finding similar to previous studies (Lucier and Egizii, 1986; Panneton, 1991; Panneton et al., 2006; Hollandsworth et al., 2009). Additionally, newly presented results show NTS labeling is present after injection of WGA into the nasal passages in rats with bilaterally cut AENs. Collectively these results indicate the AEN cannot be responsible for labeling of NTS after WGA injection into the nasal passages. This is in disagreement with Anton and Peppel (1991) who were convinced the AEN is responsible for interstitial NTS labeling. Instead, we contend NTS labeling seen after WGA injection of the nasal passages in rats must be due to other, non-AEN, nerve(s) that innervate the internal nasal passages. These other nerve(s) could be branches of the maxillary division of the trigeminal nerve, since maxillary and mandibular trigeminal nerves have projections to the solitary nucleus, (Marfurt, 1981; Jacquin et al., 1983; Hamilton and Norgren, 1984; Nomura et al., 1984; Takemura et al., 1991). Alternatively, since WGA injected into the nasal passages could have leaked posteriorly into the nasopharyngeal region, NTS labeling after bilateral AEN sectioning could be due to labeling of vagal-glossopharyngeal afferents (Contreras et al., 1982; Altschuler et al., 1989; Hayakawa et al., 2001).

A second difference in the brainstem labeling patterns was that after WGA was injected into the nasal passages of rats with intact AENs, central label was found ipsilaterally within the RVLM. In contrast, when WGA was injected into nasal passages after the AENs were sectioned bilaterally, labeling of the RVLM was absent. Additionally, after direct injection of WGA into the AEN, central projections within the RVLM were found. These projections were similar to those found previously after direct injection of WGA into the AEN of both the rat (Panneton et al., 2006; Hollandsworth et al., 2009) and muskrat (Panneton, 1991). Thus label within the RVLM (i.e., C1 and Bötzinger area) after WGA injection into nasal passages in intact animals is most likely due to labeling of the internal nasal branch of the AEN.

A third difference between the brainstem labeling patterns after nasal and AEN WGA injections was that after WGA injection into the nasal passages, central WGA label was found bilaterally along the dorsolateral edge of Sp5I adjacent to the trigeminal track at and just rostral to obex. This labeling was still found after WGA injection into the nasal passages of rats with the AENs cut bilaterally, but was not found after direct WGA injection into the AEN. Thus the labeling along the dorsolateral edge of the Sp5I is not due to labeling of the AEN, and instead is likely due to labeling of the nasopalatine nerve, as this location is labeled after WGA injections into this nerve.

A fourth difference in the brainstem labeling patterns was that after WGA injection into the nasal passages, central WGA label was found ipsilaterally, and sometimes bilaterally, as a thin strip along the dorsolateral trigeminal tract adjacent to SP5O. This labeling was still found after WGA injection into the nasal passages with the AENs cut bilaterally, but was not found after direct WGA injection of the AEN. Therefore, this labeling is not from the AEN, and instead other nerve(s) innervating the nasal passages, probably maxillary nerves, are likely responsible for this labeling pattern.

#### Non-AEN Innervation of the Internal Nasal Passages

**Figure 12:**
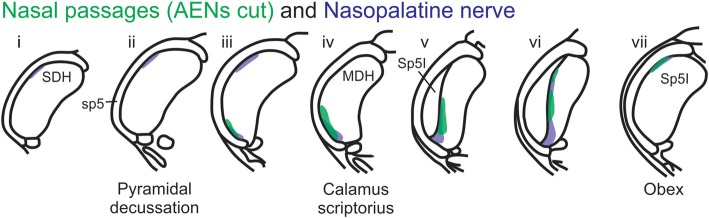
Summary of MDH labeling after WGA injection into the left nasal passages and WGA injection into nasopalatine nerve. Labeling pattern from the nasal passages with the AENs cut bilaterally (from Figure 3) is shown in transparency overlay with the labeling pattern from nasopalatine nerve (from Figure 7).

A new finding from the present research is that other nerve(s), besides the AEN, do innervate the nasal passages, and project to the ventral MDH. This conclusion was found in two ways. First, after the AENs were cut bilaterally, injection of WGA into the nasal passages resulted in a similar (albeit less prominent) pattern of labeling within the ventral tip of MDH. The nerve(s) involved likely are branches of the maxillary division of the trigeminal nerve, particularly the ganglionic branches originating from the pterygopalatine (AKA sphenopalatine) ganglion, which innervate the mucosa of the more posterior portions of the internal nasal passages and nasal cavity (Bojsen-Moller, 1975; Silver, 1992; Shankland, 2001b; Silver and Finger, 2009; Gray et al., 2016). This supposition was then verified by injecting WGA into the nasopalatine nerve, a branch of the maxillary division of the trigeminal nerve. After nasopalatine nerve injection, WGA label was found within the ventral tip of MDH, between pyramidal decussation and obex, especially along the transition with Sp5I just rostral to calamus scriptorius. The central labeling from the nasopalatine nerve almost superimposes upon labeling from the nasal passages both when the AENs are intact (Figure 11B) and cut bilaterally (Figure 12). This indicates that, in addition to the AEN, the nasopalatine nerve provides innervation of the internal nasal passages, presumably its more posterior portion (Silver, 1992; Silver and Finger, 2009).

The central projections of the pterygopalatine nerves in the cat were previously found within the rostral MDH near obex, but not at the ventral tip of MDH (Shigenaga et al., 1986). This may be because these nerves provide innervation of both the nasal passages and oral cavity (Shankland, 2001b; Gray et al., 2016). Shigenaga et al. (1986) injected the intraoral branches of these nerves, whereas we would expect the intranasal branches of these nerves to have a slightly different somatotopic representation, primarily more toward the ventral tip of MDH.

Another maxillary nerve, the ION, also provides some ventral MDH labeling after injection of WGA into the nasal passages. The ION does innervate the nasal septum, but it primarily innervates cutaneous structures, such as the skin of the side of the nose and lower eyelid, and lower and upper lip (Shankland, 2001b; Gray et al., 2016). Furthermore, the ION provides the major sensory innervation of the mystacial vibrissae of rodents (Vincent, 1913; Dykes, 1975; Jacquin et al., 1984; Rice et al., 1993; Waite and de Permentier, 1997; Maklad et al., 2004). Injection of WGA into the main trunk of the ION within the orbit labeled the superficial laminae of MDH in a fashion similar to WGA labeling of individual vibrissae (Arvidsson, 1982; Nomura et al., 1986; Arvidsson and Rice, 1991) or the palpebral conjunctiva (Panneton et al., 2010c). Also, the dorso-ventral distribution of the ION WGA labeling is similar to the distribution of Fos-positive neurons seen after ION injury (Vos and Strassman, 1995; Terayama et al., 1997). Injection of WGA into the orbital ION did label the ventral MDH more caudally, but did less so along the MDH-interpolaris transition zone. Aspects of orbital ION labeling superimposes upon much of the labeling from the nasal passages (Figure 11C). In contrast, injection of WGA into the ION immediately posterior to the mystacial pad (Figure 11D) or into the external nasal branch of the ION (Figure 11E) did not. Thus, while the orbital ION could partially innervate the internal nasal passages, the external branches of the ION do not.

#### Innervation of the Distal Internal Nasal Passages

After WGA was injected more distally within the nasal passages (i.e., immediately inside the nasal vestibule and brushed superficially on the glabrous skin immediately outside the vestibule) in rats with intact AENs, the central pattern of labeling within MDH was almost identical to both: (1) application of WGA deeper within nasal passages; and (2) direct injection of WGA into the AEN. Interestingly, when the AENs were sectioned bilaterally and WGA was applied to this same location, no central terminations were observed within MDH. This strongly suggests the external nasal vestibule and glabrous skin of the nose is only innervated by the external nasal branch of the AEN and by no other ophthalmic or maxillary trigeminal nerves. It seems surprising an anatomical location such as this has afferent innervation provided by only a single nerve.

An additional finding was that in animals with intact AENs, WGA injection immediately inside the nasal vestibule did not label the RVLM. Thus the external nasal branch of the AEN does not project to the C1/Bötzinger area. This confirms AEN projections to the RVLM are solely due to labeling of the internal nasal branch of the AEN within the nasal passages.

### Pattern of Central WGA Labeling From the External Nasal Region

When WGA was injected into the hairy skin of the tip of the nose superior to the nasal vestibule, label was found within the ventral tip of MDH, caudally near the pyramidal decussation. The external nasal nerve, a peripheral branch of the AEN, innervates the superior part of the ala and apex of the nose (Greene, 1963; Shankland, 2001a; Gray et al., 2016), and thus innervates the hairy skin of the tip of the nose. However, to determine if other non-AEN nerve branches also innervate this area, the AENs were cut bilaterally in all these animals prior to WGA injection. Since WGA label appeared in MDH after the AENs had been cut bilaterally, this region is innervated by another nerve besides the external nasal branch of the AEN. This other nerve may potentially be the supratrochlear nerve, a branch of the frontal nerve from the ophthalmic division of the trigeminal nerve. The supratrochlear nerve innervates the skin of the bridge of the nose and upper eyelid (Greene, 1963; Shankland, 2001a; Gray et al., 2016). However, when WGA was injected directly into the supratrochlear nerve, label was found in the ventrolateral tip of the rostral SDH caudal to the pyramidal decussation. Thus this supratrochlear label did not match the central label seen after WGA was injected into the hairy skin of the tip of the nose, which was found more rostrally near the pyramidal decussation. Therefore, it seems unlikely the hairy skin of the tip of the nose is innervated by the supratrochlear nerve. However, another nerve besides the external nasal branch of the AEN must also innervate the superior tip of the nose. Also, after WGA was injected into the hairy skin of the tip of the nose, the label found within the ipsilateral 7th motor nucleus was probably due to the labeling of the motor nerves that innervate the tip of the nose.

When WGA was injected into the glabrous skin inferior to the nasal vestibule, label was found caudally within the lateral and dorsolateral MDH. Similar labeling was found both when the AENs were intact or cut bilaterally. Since this labeling pattern is: (1) more lateral and dorsal compared with the labeling found after injection of WGA into the AEN; and (2) nearly identical both before and after bilateral sectioning of the AENs, these findings indicate the glabrous skin inferior to the nasal vestibule is not innervated by the external nasal branch of the AEN. Sensory innervation of the glabrous skin inferior to the nasal vestibule could be provided by the external nasal branch of the ION, which projects to the skin of the nose and muzzle (Greene, 1963; Shankland, 2001a; Gray et al., 2016). Injections of WGA into either the ION posterior to the mystacial pad or the external nasal branch of the ION did show some labeling similarities compared with the central MDH projections from the glabrous skin of the nose, especially nearer to the pyramidal decussation. However, label from the external nasal branch of the ION was found more ventrally and extended more rostrally within MDH than was WGA label from the glabrous skin inferior to the nasal vestibule. This suggests that in addition to the external nasal branch of the ION, another nerve also contributes to the innervation of this region. Also, label found within the ipsilateral 7th motor nucleus in rats both with and without intact AENs was probably due to the labeling of motor nerves innervating the glabrous nose.

When WGA was injected under the skin along the bridge of the nose in rats with intact AENs, label was only found caudal to the pyramidal decussation. The pattern of labeling was nearly identical after the AENs were sectioned bilaterally. This indicates the bridge of the nose is not innervated by the external nasal branch of the AEN. The supratrochlear branch of the frontal nerve innervates skin of the bridge of the nose (Greene, 1963; Shankland, 2001a; Gray et al., 2016). Injection of WGA into the supratrochlear nerve also only labeled the spinal cord. However, after supratrochlear nerve injection, WGA label was found only in the ventral tip of, and more caudally within, SDH when compared with labeling found after injection of the skin along the bridge of the nose. Since the labeling pattern from the supratrochlear nerve does not overlap with the labeling pattern from the bridge of the nose, another nerve besides the supratrochlear nerve must also innervate the bridge of the nose. A possibility is the infratrochlear nerve, which is a branch of the ophthalmic nasociliary nerve (Greene, 1963; Shankland, 2001a; Prendergast, 2013; Gray et al., 2016). Also, label found within the ipsilateral 7th motor nucleus after these injections was probably due to WGA labeling of motor nerves that innervate muscles along the bridge of the nose.

### Central Projections to Contralateral Dorsal Horn

After application of WGA to the left internal nasal passages of rats with intact AENs, label was found in the right ventral MDH just rostral to calamus scriptorius. This contralateral projection from the nasal passages was not found in a previous study using similar methodology (Anton and Peppel, 1991). Furthermore, after application of WGA to the internal nasal passages of rats with bilaterally cut AENs, label was still found in the contralateral MDH along the MDH-interpolaris transition zone. Jacquin et al. (1990) found contralateral trigeminal projections to medullary and SDHs after injecting HRP into the trigeminal ganglion. However, they concluded centrally decussating trigeminal primary afferents do not originate in the nasal mucosa, as no contralateral labeling was found after applying HRP to the cut end of the ethmoidal nerve. We too found no contralateral labeling of the dorsal horn after direct injection of WGA into the AEN, similar to findings previously reported in both rats (Panneton et al., 2006; Hollandsworth et al., 2009) and cats (Lucier and Egizii, 1986). If contralateral projections to the dorsal horns from the internal nasal passages do not occur via the AEN, we suggest maxillary trigeminal nerves innervating the internal nasal passages are responsible for these contralateral projections. This is an interesting conclusion, as contralateral trigeminal primary afferent projections are predominantly of ophthalmic and mandibular origin (Jacquin et al., 1982, 1983; Takemura et al., 1987; Jacquin et al., 1990; Marfurt and Rajchert, 1991). While contralateral maxillary projections have been reported previously, as the cat ION projects to the contralateral C1–C2 dorsal horn (Marfurt, 1981), we did not see contralateral dorsal horn projections in the nasopalatine nerve or any of the three ION locations into which we injected WGA. Therefore, although we have no direct evidence of contralateral maxillary projections, we do suggest the ganglionic branches of the maxillary division innervating the mucosa of the internal nasal passages, particularly the midline structures of the nasal cavity, have contralateral dorsal horn projections.

After application of WGA to midline external nasal regions, label was found in both the ipsilateral and contralateral spinal and MDHs. However, after WGA was directly injected into nerves innervating external nasal regions, no label was found within the contralateral medullary or SDHs. It is likely then that both the left and right nerves that innervate these superficial external nasal regions send fine projections within the skin that cross over the center midline, which could account for the observed contralateral labeling.

### Implications for Initiation of the Mammalian Diving Response

**Figure 13:**
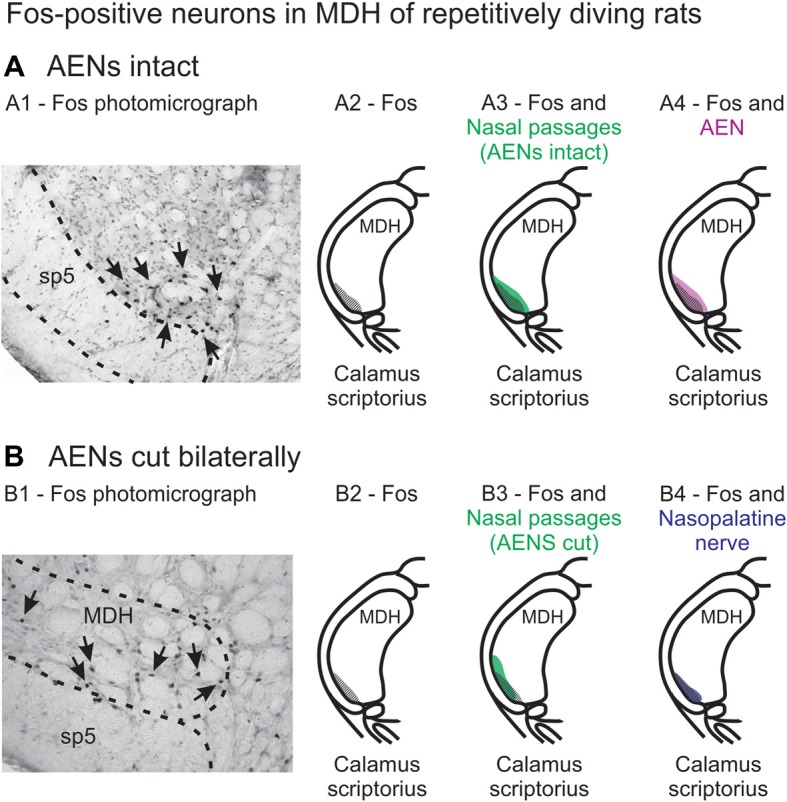
Fos-positive neurons in MDH of repetitively diving rats from **(A)** rats with intact AENs and **(B)** rats with AENs cut bilaterally. Photomicrographs in **(A1)** from McCulloch (2005) and **(B1)** from McCulloch et al. (2016) show brainstem tissue immunohistologically stained to identify Fos-positive neurons from repetitively diving rats. Rostral-caudal level is just rostral to calamus scriptorius. Arrows point to Fos-positive neurons in MDH. Cross-hatching in **(A2)** and **(B2)** indicate regions of Fos-positive neurons from photomicrographs in **(A1)** and **(B1)**, respectively. Area of Fos labeling **(A2)** is shown in transparency overlay with **(A3)** the WGA labeling pattern from the nasal passages with the AENs intact (from Figure 2Aiv) and with **(A4)** the WGA labeling pattern from the AEN (from Figure 6Aiv). Area of Fos labeling **(B2)** is shown in transparency overlay with **(B3)** the WGA labeling pattern from the nasal passages with the AENs cut bilaterally (from Figure 3Aiv) and with **(B4)** the WGA labeling pattern from the nasopalatine nerve (from Figure 7Aiv). Photomicrographs from McCulloch (2005) and McCulloch et al. (2016) are used with permission.

When animals submerge underwater, sensory information from the nose and nasal region travels into the brainstem, constituting the afferent limb of the diving response neural pathway (McCulloch, 2012; Panneton, 2013). At the first node in this pathway, the afferent information produces activation of secondary neurons within the trigeminal nucleus (Hollandsworth et al., 2009), as determined by their production of Fos protein (Coggeshall, 2005). These secondary neurons are located within the ventral tip of MDH, between pyramidal decussation and obex, peaking just rostral to calamus scriptorius with the appearance of Sp5I (Figures 13A1,A2; McCulloch, 2005; Panneton et al., 2010a, 2012; McCulloch et al., 2016). An important aim of the present study was to determine whether the afferent sensory information that initiates activation of these secondary neurons originates from the internal nasal passages or from more external nasal regions. When WGA was injected into the nasal passages of rats with intact AENs, the pattern of WGA labeling within the ventral MDH was very similar to the pattern of Fos-positive neurons from intact voluntarily diving rats (Figure 13A3). Since these two labeling patterns are so similar, the activation of secondary neurons during diving most likely results from stimulation of nerves innervating the internal nasal passages. In comparison, external nasal regions, including the hairy skin of the nose superior to the nasal vestibule, the glabrous skin of the nose inferior to the nasal vestibule, and the skin along the bridge of the nose do not have afferent projections to the parts of the ventral MDH that express Fos protein during diving. Central WGA projections from each of these regions are more caudal and/or dorsal to neurons within the ventral MDH that are activated during diving. Therefore, the present results strongly suggest afferent fibers from the internal nasal region, but not external nasal region, project to MDH in an appropriate anatomical way to cause the activation of secondary neurons within the ventral MDH that express Fos protein during diving. This appears to be different than in humans, however, where application of a cold compress to the facial region under the eyes can initiate a bradycardia similar to that observed during diving (Schuitema and Holm, 1988; Sterba and Lundgren, 1988; Andersson et al., 2004).

A second aim of the present study was to determine which trigeminal nerve(s) potentially carries the sensory information important for initiation of the diving response from the nose and nasal region into the brainstem. It had been long assumed that the AEN is primarily responsible for providing the sensory afferent signals that initiate the diving response ((McCulloch, 2012; Panneton, 2013). The present results support this postulate, as the central labeling of the AEN almost superimposes upon labeling from the nasal passages (Figure 11A), and the location of Fos-positive neurons within the MDH activated by voluntary diving (Figure 13A4; McCulloch, 2005; Panneton et al., 2012; McCulloch et al., 2016). The present results also show that a second trigeminal nerve, the nasopalatine nerve, could also provide sensory afferent signals important for initiation of the diving response. After the AENs are cut bilaterally, Fos-positive neurons still appear within the ventral tip of MDH after repetitive diving (Figures 13B1,B2). Additionally, the location of these Fos-positive neurons overlap with WGA labeling from the nasal passages after the AENs were cut bilaterally (Figure 13B3). These findings strongly suggest another nerve besides the AEN innervates the nasal passages. The central labelling of the nasopalatine nerve also almost superimposes upon labeling from the nasal passages (Figure 11B), and the location of Fos-positive neurons within the MDH activated by voluntary diving in rats with bilaterally sectioned AENs (Figure 13B4). The nasopalatine nerve’s potential ability to activate secondary neurons within MDH during voluntary diving in rats would be especially relevant during voluntarily diving after the AENs were sectioned bilaterally (Chotiyanonta et al., 2013; McCulloch et al., 2016). The ION is another nerve potentially providing necessary afferent innervation for initiation of the diving response (Panneton, 2013). The WGA labeling from the orbital ION showed partial overlap with WGA labeling from the nasal passages (Figure 11C), while the more peripheral branches of the ION did not (Figures 11D,E). Thus these peripheral branches of the ION probably are not involved in the initiation of the diving response. Therefore, although branches of the ION may innervate portions of the nasal passages, we feel less confident in including the ION as one of the nerves involved in the initiation of the diving response. Collectively, the present results strongly suggest the innervation of the anterior nasal passages by both the AEN and nasopalatine nerve provides the afferent information responsible for the activation of secondary neurons within MDH during voluntary diving in rats.

## Conclusion

In this study, using selective injections of the transganglionic tracer WGA in rats, we determined the central terminal projections of nerves innervating the external nose, nasal vestibule, and nasal passages, and compared that to the central terminal projections from the internal nasal passages. Central projections from the nasal passages were found prominently within the ventral tip of MDH, between pyramidal decussation and obex, especially along the transition with Sp5I just rostral to calamus scriptorius. This pattern of labeling was very similar to when WGA was directly injected into either the AEN or nasopalatine nerve. Therefore the present results strongly indicate that the internal nasal branch of the AEN and the nasopalatine nerve both provide primary innervation of the internal nasal passages. There were, however, several differences noted in the central labeling pattern of the brainstem when WGA was injected into the nasal passages vs. directly into the AEN. Projections from the nasal passages to the contralateral MDH were also found. Regarding the external nasal regions, the tip of the nose superior to the nasal vestibule is innervated by neither the external nasal branch of the AEN nor the supratrochlear nerve; the glabrous skin inferior to the nasal vestibule is not innervated by the external nasal branch of the AEN, but could be partially innervated by the external nasal branch of the ION; and the skin along the bridge of the nose is innervated by neither the external nasal branch of the AEN nor the supratrochlear nerve.

In conclusion, the present results strongly suggest afferent fibers from the internal nasal passages, but not external nasal region, project to MDH in an appropriate anatomical way to cause the activation of secondary neurons within the ventral MDH that express Fos protein during diving. Therefore, both the internal nasal branch of the AEN and the nasopalatine nerve, but not the ION, are likely to provide the sensory information responsible for the activation of secondary neurons within MDH during initiation of the mammalian diving response in rats. This appears to be different than in humans, where application of a cold compress to the external facial region can initiate the diving response.

## Author Contributions

All authors had full access to all data in the study and take responsibility for the integrity of the data and the accuracy of the data analysis. PM mentored, designed, coordinated the study, and drafted the manuscript. KL and BD acquired and analyzed data. KD acquired data and coordinated data analysis. All co-authors contributed to manuscript preparation and editing.

## Conflict of Interest Statement

The authors declare that the research was conducted in the absence of any commercial or financial relationships that could be construed as a potential conflict of interest.

## Abbreviations

AEN: anterior ethmoidal nerve
ION: infraorbital nerve
MDH: medullary dorsal horn
NTS: nucleus tractus solitaries
RVLM: rostral ventrolateral medulla
SDH: spinal dorsal horn
sp5: spinal trigeminal tract
Sp5I: spinal trigeminal nucleus interpolaris
Sp5O: spinal trigeminal nucleus oralis
WGA: wheat germ agglutinin.
